# Boosting visual cortex function and plasticity with acetylcholine to enhance visual perception

**DOI:** 10.3389/fnsys.2014.00172

**Published:** 2014-09-18

**Authors:** Jun Il Kang, Frédéric Huppé-Gourgues, Elvire Vaucher

**Affiliations:** ^1^École d’optométrie, Université de MontréalMontréal, QC, Canada; ^2^Département de Neuroscience, Université de MontréalMontréal, QC, Canada

**Keywords:** attention, cholinergic system, cognitive enhancement, cortical plasticity, nicotinic receptors, muscarinic receptors, perceptual learning, visual cortex

## Abstract

The cholinergic system is a potent neuromodulatory system that plays critical roles in cortical plasticity, attention and learning. In this review, we propose that the cellular effects of acetylcholine (ACh) in the primary visual cortex during the processing of visual inputs might induce perceptual learning; i.e., long-term changes in visual perception. Specifically, the pairing of cholinergic activation with visual stimulation increases the signal-to-noise ratio, cue detection ability and long-term facilitation in the primary visual cortex. This cholinergic enhancement would increase the strength of thalamocortical afferents to facilitate the treatment of a novel stimulus while decreasing the cortico-cortical signaling to reduce recurrent or top-down modulation. This balance would be mediated by different cholinergic receptor subtypes that are located on both glutamatergic and GABAergic neurons of the different cortical layers. The mechanisms of cholinergic enhancement are closely linked to attentional processes, long-term potentiation (LTP) and modulation of the excitatory/inhibitory balance. Recently, it was found that boosting the cholinergic system during visual training robustly enhances sensory perception in a long-term manner. Our hypothesis is that repetitive pairing of cholinergic and sensory stimulation over a long period of time induces long-term changes in the processing of trained stimuli that might improve perceptual ability. Various non-invasive approaches to the activation of the cholinergic neurons have strong potential to improve visual perception.

## Introduction

Boosting the brain’s functioning during rehabilitation paradigms might help individuals with cognitive or sensory deficits to better recover their abilities. In this review, we will examine how the cholinergic system might help in this regard by specifically focusing on visual function. Recent knowledge about the cellular and functional organization of the primary visual cortex (V1) is particularly interesting for the deciphering of the neurobiological mechanisms of perceptual learning and its modulation by the cholinergic system. V1 is the first cortical step of the integration of complex visual stimuli and is decisive in the selection of specific stimuli from the visual field. This process further orients processing in higher cognitive cortical areas involved in elaboration of fine visual conscious perception. Thus, cholinergic modulation of visual processing in V1 would have strong effects on the fine-tuning of perception and the acquisition of memory traces.

Perceptual learning is the long-term improvement of the ability to detect or discriminate specific sensory stimuli without interfering with or diminishing other skills that results from training over a sustained period of time (Fahle and Poggio, 2002; Fahle, 2009; Roelfsema et al., 2010). In vision, improvements in the discrimination of specific attributes of a stimulus, such as its orientation (Ramachandran and Braddick, 1973; Fiorentini and Berardi, 1980; Mayer, 1983), contrast (Hua et al., 2010) or vernier acuity (McKee and Westheimer, 1978), have been demonstrated using such paradigms. Increases in visual capacity should go together with increases in the numbers of neurons that encode the trained stimulus in the V1 and the expansions of the cortical maps that represent the stimulus (Kilgard and Merzenich, 1998). The signal-to-noise ratio is usually increased. The connectivity between neurons and efficiency of the neuronal transmission, i.e., the strength of the input they transmit as well as the short processing time, should also be increased. Changes in dendritic spines number, morphology and synaptic plasticity (i.e., long-lasting modifications of the strength of the post-synaptic electrical signal) have also been demonstrated during perceptual learning (Gilbert and Li, 2012). However, it should be assumed that the neurons involved in perceptual learning increase the amount of information that they carry while preserving their primary selective response properties (Gilbert et al., 2001). Perceptual learning is also facilitated either by attention (Ahissar and Hochstein, 1993) or reinforcement by reward expectation (Seitz et al., 2009); both of these process enhance neuronal transmission efficiency.

Perceptual learning or increased cortical processing of specific stimuli is generally achieved with repetitive training. It has been recently suggested that it can also be boosted by neuromodulation and extrinsic control of the cerebral neuromodulatory systems by electrical or pharmacological means. The cholinergic system, which uses acetylcholine (ACh) as a neurotransmitter, is particularly relevant because it widely innervates V1 and alters the efficiency of neurons. The injection of ACh or its analogs into V1 has been shown to increase neuronal responses and trigger synaptic plasticity (Gu, 2003) and cortical plasticity (Bear and Singer, 1986). More specifically, the administration of ACh during visual processing increases thalamocortical input while reducing intracortical recurrence (Gil et al., 1997; Disney et al., 2007; Soma et al., 2013a) and thus enhances specific stimulus processing and output. This diversity of the actions of ACh is due to the ubiquitous localization of both ionotropic nicotinic receptors (nAChRs) and metabotropic muscarinic receptors (mAChRs) in V1 (Levey et al., 1991; Disney et al., 2006; Amar et al., 2010), which are involved in the facilitation of cortical activity and synchronized cortical activity. In addition to the direct and acute effects of ACh, an increasing number of studies have recently shown that repetitive cholinergic activation of the visual cortex has also the ability to enhance visual perception. The repetitive pairing of ACh release with exposure to a visual stimulus improves several visual capacities, such as contrast sensitivity (Mayer, 1983; Hua et al., 2010), motion detection (Rokem and Silver, 2010), working memory (Furey et al., 2000; Bentley et al., 2004), texture discrimination (Beer et al., 2013) and visual acuity (Kang et al., 2014) in both humans and animals. Many animal studies have also demonstrated the involvement of the cholinergic system in perceptual learning in different sensory modalities, including olfaction (Wilson et al., 2004) and audition (Bakin and Weinberger, 1996). These improvements suggest that paired visual and cholinergic stimulation induces perceptual learning possibly via synaptic and cortical modifications linked to attention mechanisms (Herrero et al., 2008) or reward expectation (Chubykin et al., 2013) and cortical plasticity. The repetition of such pairings would result in a more efficient processing and increased automaticity of visual stimuli. This could be related to reduced strength of connectivity between attention regions and V1 (Ricciardi et al., 2013) and a role of ACh in perceptual inference and repetition suppression (Moran et al., 2013).

**Figure 1:**
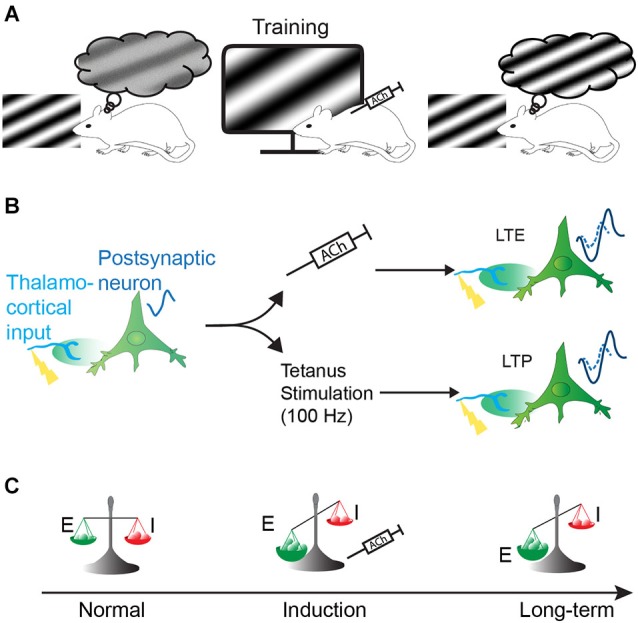
**Hypothesis of the effect of the cholinergic system on visual perception**. Increase of perceptual capacity (perceptual learning) can be obtained by naturally or artificially activating the cholinergic system during sensory training. This perceptual learning might be achieved by long-term facilitation of cortical responses and/or change of the excitatory/inhibitory balance. **(A)** Representation of the improvement of visual perception in the rat by pairing the presentation of a specific sinusoidal grating coupled to cholinergic system activation (represented by injection of acetylcholine, ACh). **(B)** Long-term enhancement (LTE) of the cortical responses by ACh (upper path) share common features with classical long-term potentiation (LTP, lower path): visual stimulation of presynaptic input evokes small responses (represented by a resulting small visual evoked potential (VEP) signal waveform) in post-synaptic neurons. If paired to cholinergic activation, the presynaptic stimulation induces a long-term enhancement of neuronal responses (upper path, represented by an increased VEP signal waveform). This mechanism is similar to LTP where theta-burst stimulation (100 Hz) in lateral geniculate nucleus (LGN) induces an increase of postsynaptic potentiation in the cortex (lower path). VEP signals are imaginary waveform to compare neuronal response magnitude, as recorded in our previous experiments. **(C)** Cortical plasticity induced by ACh could also result from a change in excitatory and inhibitory balance by changing the strength of the excitatory synapse over inhibitory synapses, resulting in long-term modification of cortical responses.

Our research hypothesis proposes that cholinergic effects in V1 contribute to perceptual learning and can thus be used to voluntarily develop one’s brain capacity and aid the restoration of visual function. In the present review, we will discuss how ACh might improve perceptual capacities, particularly during repetitive stimulation paired with visual stimulation, which are related to its roles in the long-term enhancement of cortical responsiveness and cortical plasticity (Figure 1). Specifically, we will first discuss the diverse effects of ACh on V1 neuron function and connectivity and relate these effects to the background theory of the cholinergic modulation of neural mechanisms and brain function. To assess these neuronal mechanisms, we will primarily discuss studies that have been performed in rodents and non-human primates (for more information about cholinergic effects on human cognition, see Drevets et al., 2008; Bentley et al., 2011).

## Organization of the cholinergic system in V1

**Figure 2:**
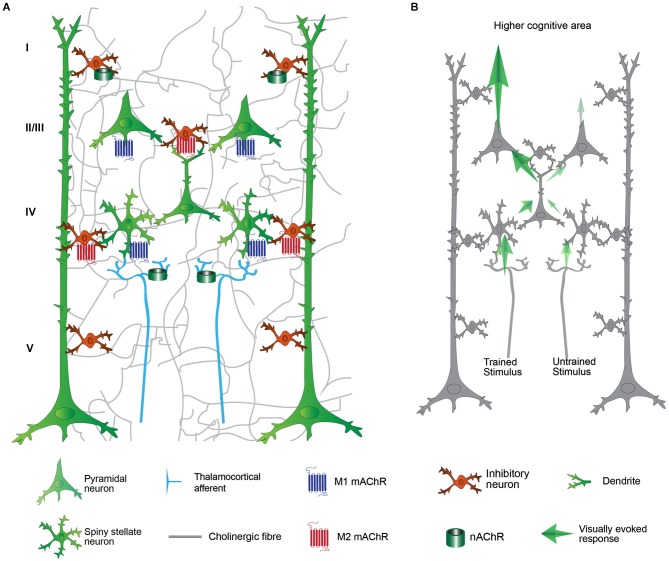
**Schematic representation of the primary visual cortex (V1) and its cholinergic modulation on cortical processing. (A)** Thalamocortical afferent (light blue fibers) from LGN conveying stimulus information reach spiny stellate neuron in the layer IV. The input is transferred to the layer II/III, then layer V and to higher visual area. The cholinergic activation modulates the visual processing in virtually all the levels of V1 connectivity by nicotinic (green cylinder) and muscarinic (seven transmembrane domains molecules) receptors. **(B)** Cortical processing after VS/HDB training. The cortical processing for the trained stimulus is significantly enhanced after VS/HDB training but un-trained stimulus processing is not affected. Note that the input from the thalamus is similar but the feedforward propagation is increased. Excitatory influences are shown in blue arrows. The strength of the response enhancement is represented by the contrast of the arrow. Layer VI and horizontal connections are omitted for clarity.

Cholinergic fibers are distributed throughout the cortical layers of V1 (Lysakowski et al., 1989; Avendaño et al., 1996; Mechawar et al., 2000), which suggests that ACh might affect every step of visual processing (Figure 2A).

### Local effect of the cholinergic fibers

The cholinergic system influences the local network by diffuse transmission rather than by synaptic transmission (Descarries et al., 1997; Yamasaki et al., 2010). This property is related to the fact that ACh is released from the varicosities that are distributed along the cholinergic axons and that these varicosities show only rare synaptic organizations at the ultrastructural level (Umbriaco et al., 1994; Vaucher and Hamel, 1995; Mechawar et al., 2000). However, the modulation of the cortex by ACh is not widespread and is primarily selective and adapted to the local microfunction due to the differential distribution of varicosities along the cholinergic axons (Zhang et al., 2011) and the differential distribution of the cholinergic receptor subtypes on different neuronal targets. Moreover, ACh release might be triggered by local neuronal activity to induce locally restricted rather than generalized action of the cholinergic system (Laplante et al., 2005). The variety of the cholinergic receptors and their distributions convey subtype-specific functions (Thiele, 2013; Groleau et al., 2014). In V1, AChRs exhibit differential subtype densities across the cortical layers (I-VI) on both excitatory (Gulledge et al., 2009; Thiele, 2013) and inhibitory neurons (Hashimoto et al., 1994). The distinct actions of cholinergic receptors can be related to differences in the conductances of the ionotropic receptor nAChRs for Na^+^, K^+^ (α_4_β_2_) and Ca^2+^ (α_7_) (Rang, 2003) and in the intracellular pathways of the different subtypes of the G-protein coupled mAChRs. Amongst the five mAChR subtypes identified, the M1, M3 and M5 mAChRs are coupled with Gq/11 proteins, which activate phospholipase C and lead to increases in intracellular Ca^2+^ and the M2 and M4 mAChRs are bound with Gi protein that inhibits adenylyl cyclase, which leads to a decrease in cAMP, the inhibition of voltage-gated Ca^2+^ channels and an increased K^+^ efflux (Caulfield and Birdsall, 1998; Wess, 2003). In addition, M1 promotes the opening of NMDARs and induces LTP in the hippocampus (Buchanan et al., 2010; Giessel and Sabatini, 2010).

### Cholinergic fibers activation in V1

Stimulation of the cholinergic system in V1 can be achieved via the administration of ACh analogs (e.g., carbachol), cholinergic receptor agonists (e.g., nicotine and selective mAChR drugs) or cholinesterase inhibitors or through electrical or optogenetic stimulation of the cholinergic neurons that project to V1. The cholinergic neurons that project to V1 are located in the basal forebrain (BF), particularly the ventral pallidum, substantia innominata and the horizontal limb of the diagonal band of Broca (HDB; Gaykema et al., 1990; Laplante et al., 2005). Although the nucleus basalis magnocellularis is the main cholinergic nucleus of the BF which innervates the cortical mantle, it projects only weakly to V1 (Luiten et al., 1987; Vaucher and Hamel, 1995); nevertheless, some studies report that the stimulation of this nucleus might induce functional changes in the visual cortex (Goard and Dan, 2009; Pinto et al., 2013). Moreover, although there are GABAergic neurons in the BF, many studies have confirmed that the effects of BF stimulation are identical to those of intracerebral injections of ACh agonists and are primarily mediated by the cholinergic fibers (Dauphin et al., 1991; Ma and Suga, 2005; Dringenberg et al., 2007; Kocharyan et al., 2008; Kang and Vaucher, 2009). There are also intrinsic cholinergic neurons that represent only 10–15% of the total cortical innervation (Eckenstein et al., 1988; Chédotal et al., 1994), and the involvement of these neurons in cortical processing remains unclear.

## Acetylcholine modulates the flow of visual information in V1

**Figure 3:**
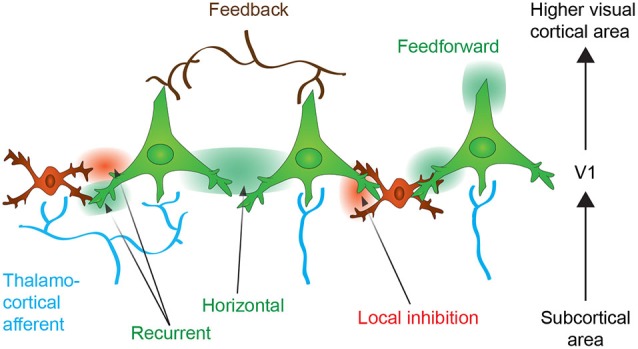
**Neuronal connectivity within the primary visual cortex (V1)**. Neurons from V1 receive thalamocortical (in blue) and corticocortical inputs originating from upper cortical areas (feedback control, in brown). The thalamocortical information is integrated within V1 and further transmitted to upper cortical areas (feedforward transmission). The activation of neurons might enhance activation or inhibition of neighboring neuron by the horizontal connections or through the local inhibitory interneurons. Recurrent connections auto-regulates neuronal activity (see text for more details). Excitatory effect is expressed as green color and inhibitory effect as red.

The efficiencies of the cortical inputs and outputs are altered by the different cholinergic receptors in both the glutamatergic and GABAergic systems according to the cortical layer, neuron and receptor subtype reached by ACh (Figure 2). V1 integrates visual information via different pathways that include the following: the feedforward thalamocortical pathways, V1 intracortical connectivities, and the feedback influence from higher cortical areas (Figure 3). The visual information arriving to layer IV of V1 from the lateral geniculate nucleus (LGN) is considered to be the dominant thalamocortical visual pathway. In contrast, the intracortical pathway might arise from neighboring neurons, local recurrent axons or more broadly from horizontal networks. The cholinergic system induces facilitation, suppression or does not affect the visual cells. Direct local effects of ACh might be opposed to the indirect effects of ACh due to neuronal interactions across layers. The general picture of the cholinergic influence on V1 is that the response to a stimulus is increased by cholinergic modulation in the thalamocortical pathway while the intracortical influence is suppressed. The cholinergic influence described in the following paragraph represents the acute effects in V1 that can participate in attention and trigger perceptual learning. The effects of the cholinergic system on long-range corticocortical relationships are also of interest but are beyond the scope of this review.

### Cholinergic modulation of thalamocortical inputs

**Figure 4:**
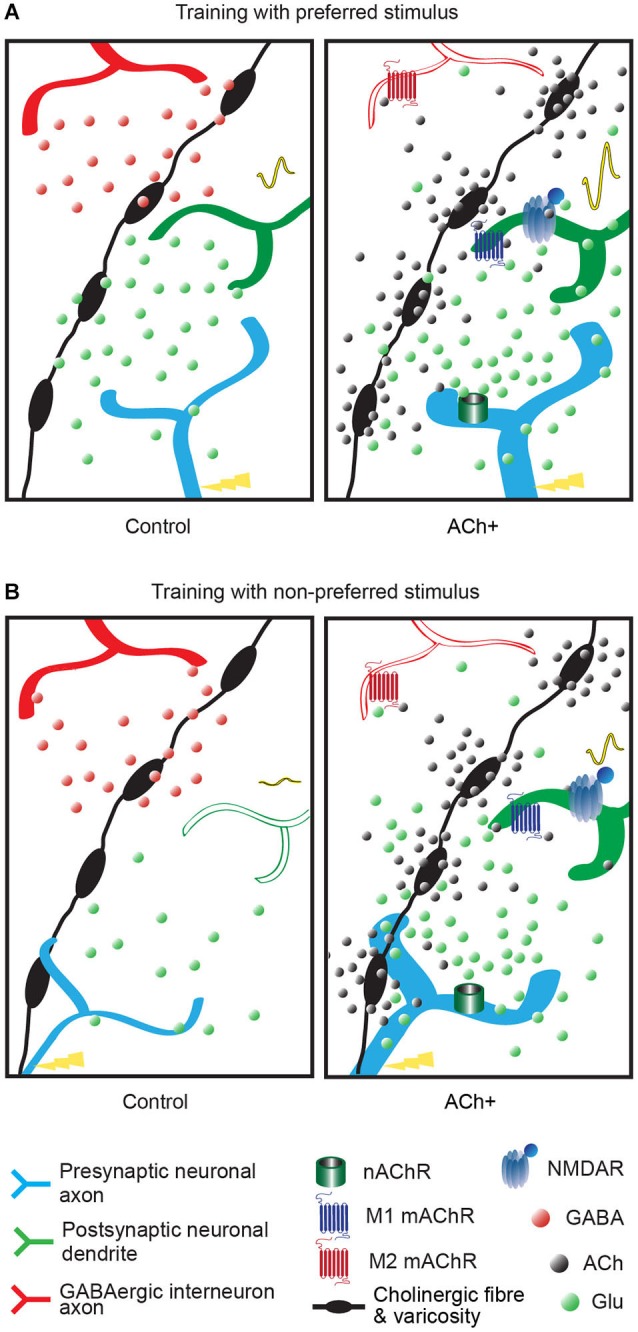
**Summary of the effect of acetylcholine on neuronal transmission of the visual inputs**. The varicose cholinergic fiber (black fiber with swellings) can act on excitatory input (blue axon), neighboring GABAergic inhibitory input (red axon) and on V1 neurons (green dendrite). Excitatory/inhibitory influences are represented by red and green dots, respectively. Cholinergic activation (ACh+, right panel) is represented by black dots. The cortical response to the stimulus is represented by a VEP signal waveform which changes are elicited by increased numbers of neurons responding to the trained stimulus or increased neurons efficiency. **(A)** Response of the V1 neuron after a training with preferred stimulus coupled to cholinergic activation (right panel, ACh+) or without (left panel, control). The cortical response to this stimulus is increased (high VEP signal waveform in right panel compared to small VEP signal waveform in left panel). In presence of cholinergic activation the inhibitory influence is reduced by M2 muscarinic receptors (mAChRs), the postsynaptic excitatory influence is increased by M1 mAChRs located on the postsynaptic neuron and nAChRs located on the thalamocortical fiber and a long-term effect is triggered by NMDA receptor activation, compared to normal condition (control, left panel). In a normal visual process (control) local or recurrent inhibition via GABAergic interneuron (in red) blocks the development to a long-term modification. **(B)** Response of the V1 neuron after a training with non-preferred stimulus coupled to cholinergic activation (right panel, ACh+) or without (left panel, control). The neuronal response to this stimulus is increased (small VEP signal waveform in right panel compared to flat VEP signal waveform in left panel). In normal condition (control, left panel), non-preferred orientation stimulus does not evoke activation in postsynaptic neurons in V1. Weak thalamocortical innervation is suppressed by GABAergic inhibition and hence fails to transmit to postsynaptic neuron. Acetylcholine can amplify the weak presynaptic input (ACh+) by nicotinic receptors and activates postsynaptic neuron through M1 muscarinic receptor. GABAergic inhibition is suppressed by M2 muscarinic receptor and NMDA receptor opening occurs leading to long-term modification.

Cortical responses to sensory stimuli transmitted by the LGN are amplified during learning and experience-dependent plasticity to emphasize relevant information (Sarter et al., 2005; Wang et al., 2013). These thalamic afferents are of prime relevance because they define the receptive fields and other properties of V1 neurons. Complex information is extracted according to its properties (e.g., orientation) via projections to different columns (in primates) or specific cells (in rodents). Cholinergic activation in this layer induces a general increase in responsiveness regardless of the features of the visual stimuli (e.g., orientation; Disney et al., 2012), which allows the cortex to respond reliably to weak stimulation (Disney et al., 2007). ACh increases the thalamocortical input through presynaptic nAChRs on the thalamocortical fibers (Gil et al., 1997; Disney et al., 2007; Figures 2A, 4). The M1 mAChR also amplifies the spiny stellate cell/pyramidal cell response through a postsynaptic intracellular pathway (Gu, 2003), but inhibition through the M4 mAChR has also been observed on spiny neurons in the somatosensory cortex (Eggermann and Feldmeyer, 2009). Interestingly, the cholinergic facilitation of thalamocortical inputs in sensory cortex slices is ACh-concentration dependent. High doses of ACh enhance the thalamocortical afferents both *in vitro* and in computational models (Hasselmo, 2006; Deco and Thiele, 2011). Together, these results indicate that, under conditions of high levels of ACh release, the enhancement of the thalamocortical inputs in layer IV facilitates the transmission of sensory information and induces experience-dependent plasticity (e.g., learning).

### Cholinergic modulation of intracortical interactions

In addition to the enhancement of thalamocortical inputs, ACh might modulate intracortical connectivity either by suppressing lateral inhibition (Kimura and Baughman, 1997; Metherate et al., 2005; Metherate, 2011) or suppressing the spread of the excitation of thalamic inputs (Kimura et al., 1999; Silver et al., 2008). The presynaptic mAChRs that are located on the glutamatergic fibers induce a suppression of the intracortical neurons (Gil et al., 1997), although the inhibition of GABAergic terminals induces a disinhibition of the pyramidal cells (Ji and Dani, 2000; Christophe et al., 2002; Seeger et al., 2004; Salgado et al., 2007). Intracortical connectivity modulates the response intensity and the output of V1 neurons (Figure 3). The lateral connections also synchronize the firing of similar neuronal populations (Gilbert and Wiesel, 1989; Lien and Scanziani, 2013), which allows for lateral correlation between neurons with similar orientation preferences during typical perceptual learning tasks (e.g., the Vernier acuity test) (Ramalingam et al., 2013). The differential action of ACh on lateral connections might simultaneously enhance specific modules of the same orientation (lateral correlation) while depressing adjacent irrelevant modules (McGuire et al., 1991; Stettler et al., 2002). A recent study using optogenetics showed that inhibition of the intracortical excitatory neurons leads to a receptive field reduction (Li et al., 2013), and this finding is consistent with the effect of ACh release in V1 (Roberts et al., 2005; Zinke et al., 2006) and the increases in the population receptive fields of M1/M3 mAChR knock-out mice (Groleau et al., 2014). Furthermore, an ACh esterase inhibitor reduces surround suppression in a perceptual study in humans (Kosovicheva et al., 2012), which could be indicative of a weakening of lateral connections. Hasselmo (2006) proposed that high ACh levels suppress the magnitude of feedback excitation, whereas low ACh levels result in weaker afferent input to the cortex. Similarly, Deco and Thiele (2011) also proposed that high ACh levels decrease the intracortical interactions and that low ACh increase these interactions. The hypothesis of these authors was confirmed in an *in vitro* study that showed that the enhancement of the recurrent cortical activity in low-dose ACh conditions was independent of the thalamocortical input (Wester and Contreras, 2013). Together, these results suggest that during intense ACh release, the intracortical connections are inhibited, which relieves the sensory cortices from recurrent connections. However, in low concentration of ACh situations, the lateral connections might amplify the thalamocortical activity amongst similarly tuned neurons.

These effects have primarily been recorded within layer II/III; however, in layers I, V and VI, which are primarily involved in feedback mechanisms, ACh might also influence feedforward processing by interacting with neurons in layers IV and II/III (De Pasquale and Sherman, 2012). Layer I neurons are densely innervated by the cholinergic projections (Vaucher and Hamel, 1995; Mechawar et al., 2000). It has been shown that inhibitory actions mediated by AChRs can suppress layer II/III (Zinke et al., 2006; Alitto and Dan, 2012; Soma et al., 2013b) and layer V pyramidal neuron activity (Lucas-Meunier et al., 2009; Amar et al., 2010) and can also inhibit the cortical GABAergic network and thus result in the disinhibition of the majority of the cortical layers (Christophe et al., 2002). It has been observed that local ACh application primarily suppresses the activity of layer VI neurons (Disney et al., 2012), which can alter the activation of all of the layers of V1 in a linear manner via the intracortical pathway (Olsen et al., 2012) and alter the activation of the thalamocortical fibers (Cudeiro and Sillito, 2006; Sillito et al., 2006). Cholinergic action might thus disinhibit the activities of other layers by suppressing layer VI. Topical injections of ACh into layer V produce the predominant effect of facilitation of the regular and fast-spiking cells (Soma et al., 2013b), although local ACh activation seems to decrease excitatory drive through presynaptic M1 mAChRs (Kimura and Baughman, 1997) and to increase inhibitory drive through M3 mAChRs (Amar et al., 2010). Similarly, an increase in the activation of GABAergic neurons activation in layer V has been observed following repetitive BF/visual pairing (Kang et al., 2014). Layer V pyramidal neurons send dense projections to the superior colliculus and diverse thalamic nuclei that are involved in focused attention.

Finally, ACh can promote the co-activation of different cortical areas and layers which might be an efficient method for the selection of visual information via a summation of the temporally coincident presynaptic spikes (Fries et al., 2007). It has been shown that visually driven gamma power is differentially distributed across the layers of V1 (Xing et al., 2012) and that gamma oscillations can be induced by cholinergic stimulation (Rodriguez et al., 2004; Bhattacharyya et al., 2013).

In conclusion, BF stimulation that facilitates the release of ACh in multiple layers of V1 might act in diverse manners and results in the enhancement of visual stimulus-driven responses. The pre-amplified responses of layer IV are filtered out by GABAergic neurons of layer II/III to transfer task-relevant information to higher visual cortical areas. The activated synaptic connections can be modulated by layers V and VI or by the feedback mechanism of layer I. Differential responses across layers might be integrated by the synchronization of their activities in the gamma-band to facilitate visual processes.

## Cellular effects of acetylcholine in V1-related attention

Most of these cellular mechanisms contribute to attentional mechanisms in V1. Attention increases the cortical response to stimuli (i.e., the signal) while lowering interference from the background (i.e., the noise). Several animal studies have described deficits of attention following cholinergic lesions or injections of cholinergic antagonists (Voytko et al., 1994; McGaughy and Sarter, 1998, 1999) and ACh has been shown to be involved in attention in V1 (Herrero et al., 2008). However, ACh release promotes rather than initiates attention. Because ACh-mediated attention and perceptual learning have crucial effects on each other, the role of ACh during visual attention is delineated in the following section to better understand how ACh enhances cortical functioning.

### Cholinergic involvement in bottom-up and top-down attention

ACh has been suggested to control the balance between bottom-up and top-down processing through attentional mechanisms (Yu and Dayan, 2002, 2005; Sarter et al., 2005). This influence is mediated by pre-synaptic thalamocortical nAChRs (Gil et al., 1997; Disney et al., 2007). Attention that is prompted by the properties of a stimulus, i.e., the saliency of the stimulus relative to the background, is said to be bottom-up attention, whereas attention that is prompted by the voluntary direction of focus toward a specific stimulus is defined as top-down attention. Although it can be difficult to separate bottom-up and top-down attentional control (Ansorge et al., 2010; Egeth et al., 2010; Eimer and Kiss, 2010; Theeuwes, 2010), some studies have shown that cholinergic activity influences bottom-up attention. The effect of ACh on bottom-up attention might occur not only in V1 but also in early processing areas such as the thalamus. For example, the direct injection of 192-IgG saporin into the BF causes a complete loss of cholinergic projections to the neocortex but causes restricted fiber lesions when injected into V1. The injection of 192-IgG saporin into the BF but not V1 affects performance in the sustained attention task (McGaughy and Sarter, 1998, 1999). In addition, compared to controls and ex-smokers, human smokers have been shown to exhibit increased subcortical activity during an attentional task (Nestor et al., 2011). These data indicate that attentional dysfunction following cholinergic lesions might be due to the disruption of detection processes that are independent of V1. However, there is no direct evidence of cholinergic enhancement effect in bottom-up attention in human studies (Rokem et al., 2010). In contrast, there is a growing body of evidence showing that ACh is involved in top-down attention. Direct effects of ACh on attention in the visual cortex have been measured (Herrero et al., 2008; Bauer et al., 2012). Specifically, Herrero et al. provided direct evidence that ACh in V1 enhances the cortical response to an attentional demand (Herrero et al., 2008). It has also been shown that lesions to the cholinergic system impair attention performance and increase neuronal activity in the PFC upon the presentation of distractors (which trigger top-down attention) (Gill et al., 2000). Taken together, these results indicate that ACh can facilitate task-relevant learning in V1 by promoting attentional states in both top-down and bottom-up manners.

### Cholinergic modulation of response gain

Response gain modulation by ACh has frequently been observed (Disney et al., 2007; Aggelopoulos et al., 2011; Bhattacharyya et al., 2013; Soma et al., 2013a) and follows the gain control model at least in terms of the contrast-response function. Increasing thalamocortical pathway input in a context-independent manner while context-dependent intracortical suppression occurs might facilitate the transmission of information related to novel stimuli. In V1, context-dependent (i.e., increases in the maximal response) and independent (i.e., increases in the baseline response) gain control due to cholinergic effects have both been observed (80% and 20%, respectively) without any laminar bias (Soma et al., 2013b). These findings could be related to the optimization of the gain of supragranular pyramidal cells controlled by ACh which could result in the detection of novel stimuli and hence perceptual learning (Moran et al., 2013). Interestingly, gain modulation was proposed as function that underlies of attentional control (Keitel et al., 2013) and network connectivity (Haider and McCormick, 2009). The high gain that results from the amplification of the responses of excited neurons is similar to attention processes (Servan-Schreiber et al., 1990; Eldar et al., 2013) and hence facilitates learning. Taken together, these results suggest that ACh might assist in visual perceptual learning via modulation of cortical responses through gain control in both stimulus-dependent and -independent manners.

## Cellular effects of acetylcholine in V1 in relation to cortical plasticity

Learning and perceptual learning are sustained by cortical plasticity which triggers anatomical reorganization of the cortical connectivity. The cholinergic system plays also a key role in cortical plasticity. For example, the blockade of cholinergic activation via cholinergic antagonists or cholinergic fiber lesions results in robust impairment of learning in rats (Conner et al., 2003; Dotigny et al., 2008) and ocular dominance plasticity in kittens (Bear and Singer, 1986). In acute preparations, cholinergic pairing is also involved in plasticity as observed in the cat auditory cortex; the application of ACh during acoustic processing alters the receptive fields of single neurons in a tone-specific manner (Metherate and Weinberger, 1990). The pairing of cholinergic and auditory stimulation also leads to the reorganization of the cortical map (Kilgard and Merzenich, 1998); i.e., an enlargement of the representation of the specifically trained frequency. Cholinergic pairing with sensory stimulation also induces long-lasting effects on cortical responsiveness observed in both the visual cortex (Dringenberg et al., 2007; Kang et al., 2014) and the somatosensory cortex (Verdier and Dykes, 2001). Cortical plasticity is essential for the occurrence of perceptual learning (for review see Fahle, 2009), although not systematic, cholinergic-sensory paired activation would thus facilitate the induction of perceptual learning in the sensory cortices (Reed et al., 2011).

### Cholinergic modulation of long-term cortical responsiveness

At the neuronal level, ACh has been shown to contribute to cortical plasticity through both the acute and long-term modulation of synaptic responses (Sato et al., 1987; Soma et al., 2012). The impairment of learning by cholinergic antagonists is similar to the effect of blocking cortical plasticity mechanisms and LTP with NMDA receptor (NMDAR) antagonists (Morris et al., 1986; Artola and Singer, 1987; Cooke and Bear, 2010). In most situations, LTP in the visual cortex induced by high theta-burst stimulation (100 Hz) (Heynen and Bear, 2001; Dringenberg et al., 2007) has been found to be NMDAR-dependent. Interestingly, cholinergic system-induced cortical plasticity has also been found to be NMDAR-dependent (Verdier and Dykes, 2001; Dringenberg et al., 2007; Kang and Vaucher, 2009) but independent of theta-burst stimulation (Kirkwood et al., 1999; Figure 1B). Previous studies in hippocampal slices have shown that NMDAR opening during LTP induction is facilitated by mAChR activation (Buchanan et al., 2010) and administration of ACh to pyramidal neurons (Shinoe et al., 2005). Additionally, NMDAR-dependent long-term facilitation of synaptic responses is associated with ACh release in V1, and LTP is impaired in the visual cortices of mAChR knock-out mice (Origlia et al., 2006).

### Cholinergic modulation of the excitation-inhibition balance

Another contribution of the cholinergic system to cortical plasticity mechanisms in V1 is the alteration of the excitatory and inhibitory (E-I) balance (Figure 1C). The excitatory and inhibitory synaptic inputs tend to equilibrate during maturation to optimally tune the neurons according to sensory experiences (Hensch et al., 1998; Sun et al., 2010) during the critical period; i.e., the post-natal time window during which mammals visual cortices are highly plastic that terminates with the maturation of the neurons. It has been proposed that disrupting the E-I balance can re-open the critical period after maturation (Hensch, 2004). Neuromodulation can also disrupt the E-I balance and contribute to cortical plasticity. Recent studies have also demonstrated numerous examples of cortical plasticity that are modified by the inhibitory system (Hensch, 2005). The onset of the critical period is accelerated by GABAA inhibitory receptor activation (Fagiolini and Hensch, 2000; Iwai et al., 2003). Conversely, it is also possible to re-induce plasticity after the critical period by reducing the inhibitory drive via the injection of GABAA receptor antagonists (Harauzov et al., 2010). As the inhibitory system is strongly modulated by the cholinergic system through the protein Lynx1 (Takesian and Hensch, 2013), which acts as a brake on nAChR-dependent plasticity (Morishita et al., 2010), by nAChRs (Christophe et al., 2002; Arroyo et al., 2012), or by mAChRs (Salgado et al., 2007), cholinergic activation might modulate the E-I balance and facilitate cortical plasticity in adults that would promote perceptual learning. An interaction between the cholinergic and GABAergic systems has been shown to occur following BF stimulation that increases the activation of Parvalbumin-positive (PV+) neurons through mAChRs (Dotigny et al., 2008; Alitto and Dan, 2012). Interestingly, Alitto and Dan used an optogenetic method to show that the nAChRs on vasoactive intestinal peptide-positive (VIP+) neurons and layer I neurons can inhibit excitatory and PV+ neurons (Christophe et al., 2002).

The cholinergic modulation of V1 thus promotes cortical plasticity through LTP-like long-term enhancement of synaptic responses to subsequent presentations of a visual stimulus and through control of the excitatory-inhibitory balance that regulate the strength of cortical output and internal connectivity. The cortical plasticity induced by cholinergic stimulation could transfer the acute cholinergic effect into long-term scale to produce visual precision.

## Repetitive cholinergic stimulation triggers perceptual learning

In summary, acute effects of cholinergic activation might amplify the thalamocortical response that promotes the transmission of sensory inputs. Intensive release of ACh might also inhibit intracortical interactions and relieve the internal brake on processing in the sensory cortices. Simultaneously, neurons with similar tuning characteristics (e.g., orientation) are co-activated via lateral connections to enhance the transfer of visual information. This cholinergic alternation might contribute to gain control modulation in both stimulus-dependent or and -independent manners and prioritize the processing of selected visual stimuli; this process might be linked to attention and is the first step of perceptual learning. The cholinergic activation also induces the NMDAR-dependent LTP-like long-term enhancement (i.e., cortical plasticity) and relief of the brakes on plasticity by altering the E-I balance. The repetitive coupling of visual and cholinergic stimulation results in reinforcement of all of these acute mechanisms and generate gamma-band synchronization. This would result in the consolidation of the synaptic strengths of new and existing neuronal connections, facilitation of the processing of certain thalamocortical inputs while suppressing others. It has been shown that increases in the cortical responses by expanding the number of neurons to a stimulation (via increases in the strength of the connections) would improve perceptual capacity (Anton-Erxleben and Carrasco, 2013). The repetitive cholinergic-visual stimulation would also increase the efficiency and automaticity of these selected pathways. These processes contribute to perceptual learning.

### Repetitive cholinergic stimulation promotes long-term potentiation

As mentioned above, ACh can induce NMDAR-dependent long-term modifications of postsynaptic glutamatergic neurons which are related to memory formation. The opening of the NMDAR launches a second messenger cascade and guides the expression of synaptic glutamate receptors (Regehr and Tank, 1990; Zhong et al., 2006) but also activates autoregulated kinases that confer a persistent improved response of the neuron to the stimulus. Immunohistochemistry for the c-Fos, which is an immediate early gene and also a transcription factor for synaptogenesis genes, has revealed that c-Fos is increased in layer II/III pyramidal neurons following a repetitive BF/visual stimulation (Kang et al., 2014), which may be indicative of the formation of new synapses and LTP mechanisms. Repetitive pairing of the cholinergic and visual stimulation also induces morphological reorganization, i.e., increase in the numbers of cholinergic varicosities in the proximity of the neurons that are sensitive to the orientation of the stimulus (Zhang et al., 2011). This increased number of cholinergic inputs, along with postsynaptic mechanisms, would increase and consolidate the response of the activated neurons to ameliorate its long-term efficiency. Thus repetitive cholinergic stimulation might enhance the encoding of the memory and morphological modifications.

### Repetitive cholinergic stimulation promotes stimulus selection and amplification

We suggest that selection of decisive inputs is controlled by the cholinergic system and contributes to the specific enhancement of a particular stimulus in perceptual learning. Modulation of the orientation selectivity of the neurons provides a great example of the possible improvement of perceptual sensitivity. Training of the rat to a preferred or a non-preferred orientation might increase the cortical response for this orientation (Cooke and Bear, 2010; Figure 4). These mechanisms are facilitated by repetitive cholinergic activation, which improve orientation discrimination of human or rats (Rokem and Silver, 2010; Kang et al., 2014). Repetitive cholinergic stimulation coupled with a certain orientation stimulus might favor the discrimination of this stimulus by two different cellular mechanisms (Figure 4). First, ACh can harmonize the activation of the whole dendritic tree of layer II/III neurons to preserve their orientation selectivity and confer responsiveness to new orientation—the dendrites of the layer II/III neurons receive inputs randomly over all of their branches, some of which are selective for the neurons’ un-preferred orientations (Jia et al., 2010). Second, the cholinergic system can enhance orientation discrimination through its interaction with the GABAergic system which assists in the sharpening (Isaacson and Scanziani, 2011) of the convergent input in the layer II/III neurons (Nassi and Callaway, 2009) but also filters out task-relevant information during perceptual learning (Roberts and Thiele, 2008). PV+ and somatostatin-positive (SOM+) GABAergic neurons are particularly involved in orientation tuning in V1 (Atallah et al., 2012; Wilson et al., 2012). It has been shown that the specific activation of PV+ neurons in V1 improves orientation discrimination abilities in awake rats during perceptual learning (Lee et al., 2012) and repetitive coupling of ACh to visual stimulation activates the V1 GABAergic neurons (Dotigny et al., 2008; Kang et al., 2014).

Thus repetitive cholinergic pairing to sensory training enhances the cortical response to trained feature of the sensory stimulus that increases the influence of the feedforward afferent.

### Repetitive cholinergic stimulation promotes perceptual learning related to attention, reward expectation and connectivity

Repetitive cholinergic stimulation first promotes attentional mechanisms that are necessary to perceptual learning (Ahissar and Hochstein, 1993; Schoups et al., 2001; Li et al., 2004; Mukai et al., 2007). These attentional processes might be also related to synchronization in the gamma band (30–90 Hz) (Fries et al., 2008) induced by repetitive cholinergic stimulation which has been proposed to facilitate the transfer of the visual information to higher visual areas. ACh can also promote task-irrelevant perceptual learning that occurs in the absence of conscious effort (Skrandies and Fahle, 1994; Watanabe et al., 2002; Gutnisky et al., 2009). Compared to task-relevant learning, which utilizes focused attention as reinforcement, studies of task-irrelevant learning have suggested that reward serves as the reinforcement signal (Seitz et al., 2009; Chubykin et al., 2013). During task-irrelevant learning, the response to a feature on which attention was not directed can also be enhanced (Watanabe et al., 2001; Giordano et al., 2009; Gutnisky et al., 2009). Interestingly, rewards can affect the visual response in V1 (Shuler and Bear, 2006), and the cholinergic system can influence reward timing expectancy (Chubykin et al., 2013). To reconcile studies showing a role of attention in perceptual learning or not, Roelfsema proposed that the attentional feedback signal related to the cholinergic system that enhances the plasticity of task-relevant features in the visual cortex also causes the inhibition of task-irrelevant features so that their plasticity is switched off (Roelfsema et al., 2010).

To a cognitive point of view, by modulating synaptic transmission in V1 and modifying the cortical dynamics, ACh can also participates in the perceptual inference to increase the strength of the representation of trained stimuli and reduce the sensory noise (Yu and Dayan, 2002) and induce sensory precision (Moran et al., 2013). It might suppress the top-down sources in the balance between top-down and bottom-up information integration in V1 (Yu and Dayan, 2005). This is in agreement with a recent study demonstrating that the cholinergic enhancement reduces the connectivity strength between cortical regions involved in attention and V1 (Ricciardi et al., 2013) and reduce the activity in frontoparietal regions (Furey et al., 2008). This suggests an increased neural efficiency in the processing of the trained stimulus that leads to an improved perceptual task performance (Ricciardi et al., 2013) linked to an automation of the cortical processing and a reduction of the attentional load required to process the trained stimulus (Furey, 2011).

Together, the findings from recent work using different techniques suggests that cholinergic pairing induces perceptual learning via different mechanisms that include the following: (1) the use of the layer II/III GABAergic system to filter the pre-amplified response from layer IV; (2) NMDAR-dependent modification at the postsynaptic level to induce long-term augmentations of individual neurons, and an increase in the numbers of cholinergic varicosities to facilitate ACh release; and (3) changes in the efficiency of the connectivity between cortical areas and bottom-up and top-down control.

## Clinical perspectives of cholinergic modulation of brain’s function

Similar with experimental data, some clinical studies have demonstrated that enhancing cholinergic system improves perception (Furey et al., 2000; Bentley et al., 2004; Wilson et al., 2004; Rokem and Silver, 2010; Beer et al., 2013; Ricciardi et al., 2013). Clinically, a method to enhance cholinergic function might involve the use of ACh esterase inhibitors, such as physostigmine, galantamine, rivastigmine or donepezil. Nicotine is also a well-known molecule that enhances cognitive function. These drugs are currently used to the treatment of Alzheimer’s disease or diverse dementia. Orally administered nicotine or smoking improve attentional performance (Nestor et al., 2011; Newhouse et al., 2011), learning (Riekkinen and Riekkinen, 1997; Olausson et al., 2004), attention (Thiel et al., 2005; Nestor et al., 2011) and memory consolidation (Beer et al., 2013) through the activation of nAChRs. Increases in ACh action due to the administration of acetylcholinesterase inhibitors or direct mAChRs agonists alleviate cognitive deficits in Alzheimer’s disease (Cummings, 2003), Parkinson’s disease (Fagerström et al., 1994; Holmes et al., 2011) and schizophrenia patients (Shekhar et al., 2008). An α7 nAChR agonist is also used as a cognitive enhancer in patients with schizophrenia (Freedman, 2013) and Alzheimer’s disease (Hilt et al., 2009). As shown in an fMRI study, cholinergic action potentiates communication efficiency between cortical areas (Wylie et al., 2012). The use of these drugs in cholinergically healthy subjects might also be beneficial for enhancing cognitive function (Buchanan et al., 2008; Demeter and Sarter, 2013).

Some pharmacological approaches have been developed to increase the perceptual learning in healthy humans. Performance improvements following the use of donepezil during a motion direction discrimination task have confirmed that systemic blockade of ACh esterase can induce perceptual learning (Rokem and Silver, 2010, 2013). Cholinergic amplifications paired with sensory stimulations might also be a promising approach to accelerating visual recovery following lesions to the retina or the optical nerve. If the neuronal mechanisms that occur during perceptual learning and after retinal lesions are similar (Gilbert and Li, 2012) (i.e., they both involve changes in the responsiveness of cortical neurons to inputs from outside the neurons’ preferred receptive fields (Darian-Smith and Gilbert, 1994)), then ACh might also aid to boost structural and functional plasticity of the visual cortex to recover from losses of retinal input.

## Conclusion

In this review, we proposed that the neuromodulator ACh, which is known for its involvement in attention and learning, might participate in and promote perceptual learning. We proposed that, via the inhibition of intracortical feedback, ACh can render V1 more sensitive to incoming thalamocortical information and enhance sensory performance. During visual processing, ACh acts on different layers to amplify the encoding of weak stimuli by strengthening synaptic connectivity, which leads to behavioral improvements. Furthermore, ACh might not only facilitate task-relevant perceptual learning via attention but also facilitate task-irrelevant learning via reward reinforcement. However, much remains to be uncovered regarding whether the cholinergic system has the potential to be used as a key mechanism for improving the function of the brain and speeding rehabilitation. Specifically, because perceptual learning occurs easily under conditions of attentional control, the development of a method to improve one’s brain capacity through improved attention and cholinergic stimulation is very attractive.

## Conflict of interest statement

The authors declare that the research was conducted in the absence of any commercial or financial relationships that could be construed as a potential conflict of interest.
